# Stent-Based Retrieval Techniques in Acute Ischemic Stroke Patients with and Without Susceptibility Vessel Sign

**DOI:** 10.1007/s00062-021-01079-1

**Published:** 2021-08-31

**Authors:** Nebiyat F. Belachew, Eike I. Piechowiak, Tomas Dobrocky, Thomas R. Meinel, Arsany Hakim, Enrique A. Barvulsky, Jan Vynckier, Marcel Arnold, David J. Seiffge, Roland Wiest, Urs Fischer, Jan Gralla, Johannes Kaesmacher, Pasquale Mordasini

**Affiliations:** 1 Department of Diagnostic and Interventional Neuroradiology, Inselspital, Bern University Hospital, University of Bern, Freiburgstraße 18, 3010 Bern, Switzerland; 2 Department of Neurology, Inselspital, Bern University Hospital, University of Bern, Bern, Switzerland; 3 Department of Diagnostic, Interventional and Pediatric Radiology, Inselspital, Bern University Hospital, University of Bern, Bern, Switzerland

**Keywords:** MRI, Clot characteristics, Stent retriever, Thrombectomy, Reperfusion

## Abstract

**Background and Purpose:**

Randomized controlled trials have challenged the assumption that reperfusion success after mechanical thrombectomy varies depending on the retrieval techniques applied; however, recent analyses have suggested that acute ischemic stroke (AIS) patients showing susceptibility vessel sign (SVS) may respond differently. We aimed to compare different stent retriever (SR)-based thrombectomy techniques with respect to interventional outcome parameters depending on SVS status.

**Methods:**

We retrospectively reviewed 497 patients treated with SR-based thrombectomy for anterior circulation AIS. Imaging was conducted using a 1.5 T or 3 T magnetic resonance imaging (MRI) scanner. Logistic regression analyses were performed to test for the interaction of SVS status and first-line retrieval technique. Results are shown as percentages, total values or adjusted odds ratio (aOR) with 95% confidence intervals (CI).

**Results:**

An SVS was present in 87.9% (*n* = 437) of patients. First-line SR thrombectomy was used to treat 293 patients, whereas 204 patients were treated with a combined approach (COA) of SR and distal aspiration. An additional balloon-guide catheter (BGC) was used in 273 SR-treated (93.2%) and 89 COA-treated (43.6%) patients. On logistic regression analysis, the interaction variable of SVS status and first-line retrieval technique was not associated with first-pass reperfusion (aOR 1.736, 95% CI 0.491–6.136; *p* = 0.392), overall reperfusion (aOR 3.173, 95% CI 0.752–13.387; *p* = 0.116), periinterventional complications, embolization into new territories, or symptomatic intracerebral hemorrhage. The use of BGC did not affect the results.

**Conclusion:**

While previous analyses indicated that first-line SR thrombectomy may promise higher rates of reperfusion than contact aspiration in AIS patients with SVS, our data show no superiority of any particular SR-based retrieval technique regardless of SVS status.

**Supplementary Information:**

The online version of this article (10.1007/s00062-021-01079-1) contains supplementary material, which is available to authorized users.

## Introduction

Mechanical thrombectomy (MT) is a safe and highly effective treatment for acute ischemic stroke (AIS) in patients with large vessel occlusions [1]. Nevertheless, efforts to increase the chances of reperfusion after MT continue. Recent studies have focused on the identification of critical clot characteristics visible on imaging that might guide the choice of the best retrieval technique [2–4]. Due to the paramagnetic property of deoxygenated hemoglobin in trapped blood cells, susceptibility-weighted sequences can be used to locate thrombus material in occluded vessels after AIS, which may be seen as a distinct loss of signal within the affected vessel. This phenomenon, which is most commonly referred to as susceptibility vessel sign (SVS), is frequently observed in AIS patients with admission magnetic resonance imaging (MRI) [4–8]. Previous studies have shown that SVS is associated with successful reperfusion and favorable clinical outcome after MT [6]; however, information on the role of first-line MT techniques in AIS patients with or without SVS is scarce. Bourcier et al. [4] indicated that stent retriever (SR) thrombectomy may be superior to contact aspiration for treating patients with SVS.

We aimed to compare reperfusion success as well as periinterventional and postinterventional complication rates between patients with anterior AIS treated with first-line SR and those treated with the combined approach (COA), composed of SR thrombectomy with additional distal aspiration, depending on SVS status.

## Methods

### Inclusion Criteria

All data analyzed in this study were gathered retrospectively by reviewing a prospective stroke database that consecutively enrolled all AIS patients who underwent MT at our hospital between January 2010 and December 2018. Patients fulfilling the following criteria were included: (1) a final diagnosis of stroke in the anterior circulation, (2) susceptibility-weighted imaging (SWI) available on baseline MRI, (3) corresponding occlusion of at least one intracranial artery on digital subtraction angiography (DSA), and (4) arterial vessel occlusion treated using a SR-based retrieval technique. The SWI quality was classified as excellent (if there were no artifacts), good (in case of minor artifacts), poor (if there were major artifacts but SVS was assessable) or very poor (if SVS was not assessable due to major artifacts). The SVS was considered technically undeterminable if the thrombus was masked due to its proximity to the skull base or it was overlain by other pathologies (i.e. hemorrhage). Patients with very poor quality SWI or technically undeterminable SVS status were excluded. All stroke patients admitted to our institution are primarily scanned via MRI; however, the final decision on whether to perform MRI or computed tomography (CT) is made by the neuroradiologists and neurologists in charge on a case by case basis depending on clinical aspects and contraindications. An SWI was an inherent part of our MRI stroke protocol throughout the duration of this study. It was only omitted when it was likely that it would yield inconclusive results based on the sequences performed beforehand (i.e. artifacts due to presence of foreign objects or motion artifacts). Ethical approval was obtained prior to conducting this study. Patients included on 1 January 2015, or later gave written or oral consent regarding use of their data for research. The need for consent was waived according to national law and regulations of the local ethics committee for patients included before this date.

### Analysis of Clinical Information

Information on the following demographics, baseline characteristics, clinical data, and cardiovascular risk factors was collected: age, sex, history of stroke, medication before AIS (antiplatelet therapy, anticoagulants, statins), hypertension, diabetes mellitus, dyslipidemia, and smoking habits. In addition, we recorded blood pressure (systolic and diastolic), glucose levels and the National Institutes of Health Stroke Scale (NIHSS) on admission, and stroke subtypes according to trial of Org 10,172 in acute stroke treatment (TOAST) classification. Also, intravenous thrombolysis prior to imaging (transfer patients) and prior to MT, time from symptom onset/last seen well to admission, time from symptom onset/last seen well to MT, and time from groin puncture to reperfusion were documented.

### Technical Information on MRI

The SWI was acquired on a 1.5 T or 3 T MRI scanner (1.5 T: MAGNETOM Avanto or MAGNETOM Aera; 3 T: MAGNETOM Verio; Siemens Healthcare AG [Erlangen, Germany]). Repetition time, echo time, flip angle, slice thickness, and intersection gap for each scanner are listed in the supplement (Supplementary Table 1).

### Imaging Analysis

**Fig. 1:**
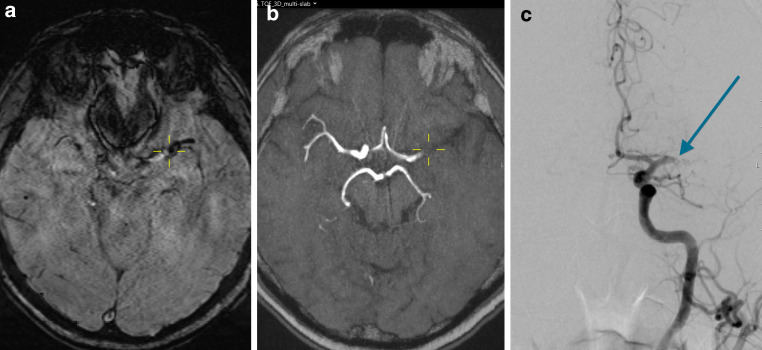
A patient with acute ischemic stroke and complete occlusion of the left middle cerebral artery (MCA) main trunk (M1 segment). SVS visible on SWI (**a**) as signal loss along the main trunk of the MCA representing the occlusive thrombus. Vessel occlusion is also seen on arterial time-of-flight sequence (aTOF; **b**) and on digital subtraction angiography (**c**). *Yellow crosshairs* are centered on the proximal end of the vessel occlusion on SWI (**a**) and on aTOF (**b**). The *blue arrow* points to the proximal end of the vessel occlusion on digital subtraction angiography (**c**)

**Fig. 2:**
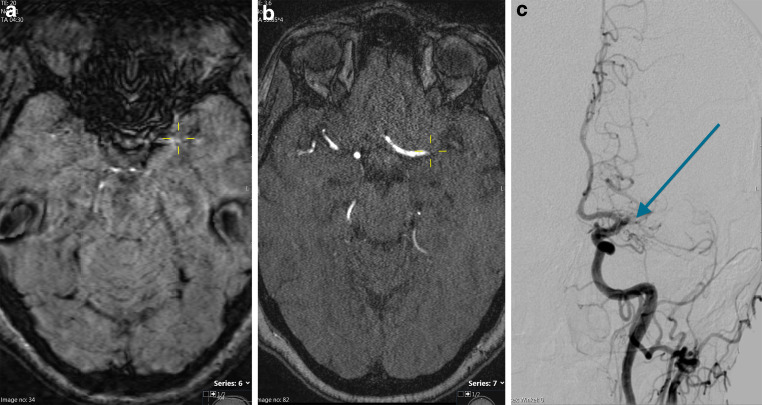
A patient with acute ischemic stroke. Susceptibility-weighted imaging (SWI) shows no SVS (**a**). Complete proximal occlusion of the left middle cerebral artery (MCA) main trunk (M1-segment) is seen on arterial time-of-flight sequence (aTOF; **b**) and on digital subtraction angiography (**c**). *Yellow crosshairs* are centered on the proximal end of the vessel occlusion on SWI (**a**) and aTOF (**b**). The *blue arrow* points to the proximal end of the vessel occlusion on digital subtraction angiography (**c**)

Two independent neuroradiologists (N.F.B. and E.A.B.), with 5 and 4 years of experience, assessed SVS retrospectively. They were blinded to all outcome parameters and had no role in patient treatment. An SWI showing a distinct signal loss corresponding to an acutely occluded, symptomatic, intracranial artery was classified as SVS⊕ regardless of its diameter compared to that of the contralateral artery (Fig. 1) if there were no alternative explanations for the signal loss observed (i.e. neighboring vein, petechial hemorrhage or microcalcification in the neighboring parenchyma). An SWI showing no SVS was classified as SVS⊝ (Fig. 2). DWI-ASPECTS (diffusion-weighted imaging Alberta stroke program early CT score) was evaluated on diffusion-weighted imaging. In addition, MRI field strength and time from symptom onset to imaging was documented for each patient.

### Digital Subtraction Angiography and Mechanical Thrombectomy

Conventional angiography was used to determine the primary site of intracranial occlusion as well as the presence of additional occlusions upstream. Experienced interventional neuroradiologists performed all MTs according to the current clinical practice guidelines and institutional protocols. First-line retrieval technique was documented as follows: stent retriever only (SR) or COA [= SR plus distal aspiration]. Table 1 lists all the first-line stent retriever devices used to treat the patients in this study. All distal aspiration catheters are listed in Supplementary Table 2. The additional use of a BGC was documented separately. The expanded thrombolysis in cerebral infarction (eTICI) score [9] was documented after the first pass and at the end of the procedure. Also, the total number of passes performed during MT was recorded. Reperfusion was considered successful if eTICI was 2B or better. A research fellow with 3 years of experience (J. K.) screened all angiography images for embolization into previously unaffected (= new) territories (ENT) and periinterventional complications (vasospasm, dissection and/or perforation).

**Table 1:** List of stent retriever devices used in this study

Device name	Size (mm)^a^	Number of patients treated
Solitaire FR (Medtronic, Irvine, California, USA)^b^	4 × 20/6 × 30/4 × 40	425
Catch (Balt, Montmorency, France)^b^	3 × 15/4 × 20/5 × 35	35
Mindframe (Medtronic, Irvine, California, USA)^c^	3 × 15	21
Trevo Provue (Stryker, Kalamazoo, Michigan, USA)^c^	4 × 30/6 × 25	10
Embotrap (Nauravi, Galway, Ireland)^c^	5 × 21	5
Preset LT (Phenox, Bochum, Germany)^b^	4 × 20	1

^a^For each device, the first value is the nominal diameter and the second value is the usable stent length expressed in mm

^b^Incomplete axial section device

^c^Complete axial section device

### Outcome

The NIHSS was evaluated a second time at 24 h after MT by a neurologist, whereas the modified Rankin scale (mRS) and mortality were assessed at 90 days after treatment by a neurologist or a certified study nurse. We defined early neurological recovery as a ≥ 4-point decrease of NIHSS 24 h after treatment compared to admission. Patients with mRS ≤ 2 at 90 days after treatment were considered functionally independent. Symptomatic intracerebral hemorrhage within 48 h after MT was assessed according to the European Cooperative Acute Stroke Study (ECASS II) definition [10].

### Statistical Analysis

SPSS software (Version 25.0; IBM, Armonk, NY, USA) was used to perform statistical analyses. Continuous variables were compared with the Mann-Whitney U‑test and categorical variables with the χ^2^-test. The association of first-pass reperfusion (FPR), overall reperfusion, thrombectomy-related complications (i.e., peri-interventional complications, ENT, and symptomatic intracerebral hemorrhage), and early neurological recovery with SVS and first-line retrieval technique was examined using multivariable binary logistic regression models. Adjustment was done for all cofactors with *p* < 0.15 as well as additional cofactors that are known or suspected to influence those parameters (additional base cofactor for all outcome variables: age, sex, bridging therapy, previous stroke, stroke subtype; additional base cofactor for thrombectomy-related complication parameters: successful reperfusion; additional base cofactor for early neurological recovery: pre-stroke mRS > 2, DWI-ASPECTS, successful reperfusion, time to reperfusion, and symptomatic intracerebral hemorrhage). Results with two-tailed *p*-values of < 0.05 were considered statistically significant and are shown as total values (*n*), percentages with respective *p*-values, medians with respective *p*-values, or adjusted odds ratio (aOR) with respective 95% CIs.

## Results

Our study identified 1317 AIS patients who underwent MT between January 2010 and December 2018. Admission MRI was available for 676 patients and SWI for 614 of them. A total of 37 patients were excluded due to very poor quality SWI or technically undeterminable SVS status. Patients with posterior circulation stroke (*n* = 44) and patients treated with contact aspiration only (*n* = 36) were also excluded. Overall, 293 were treated with SR only, whereas 204 were treated with COA. A BGC was used in 273 patients treated with SR only (93.2%) and in 89 patients treated with COA (43.6%). Acute ischemic stroke patients with admission CT instead of admission MRI had significantly higher admission NHISS, higher NIHSS at 24 h, lower reperfusion rates, lower rates of functional independence at 90 days, and higher mortality rates at 90 days (Supplementary Table 3); however, patients for whom SVS was assessable did not differ significantly from patients with nonassessable SVS with respect to demographics and core outcome parameters (Supplementary Table 4). Patients excluded due to posterior circulation stroke were significantly younger and had lower admission NIHSS than patients who were included but did not differ with respect to reperfusion and clinical outcome (Supplementary Table 5). Interrater reliability for SVS classification was strong (κ = 0.876, *p* < 0.001). All baseline characteristics and stroke-related data as well as all interventional and clinical outcome results for the SR and COA groups are provided in Tables 2, 3 and 4.

**Table 2:** Baseline characteristics and clinical data upon admission for the stent retriever (SR) and combined approach (COA) group

	Data available for (*n*/%)	All patients (*n* = 497)	SR ± BGC (*n* = 293)	COA ± BGC (*n* = 204)	P‑value
Age	497/497 (100%)	74.7 (62.6–82.0)	74.5 (62.5–82.3)	75.0 (62.5–81.5)	0.943
Sex, female	497/497 (100%)	51.7% (257)	52.6% (154)	50.5 (103)	0.650
*Risk factors*					
Hypertension	497/497 (100%)	66.6% (331)	66.2% (194)	32.8% (67)	0.826
Smoking	497/497 (99.8%)	26.4% (131)	28.3% (83)	23.5% (48)	0.224
Diabetes mellitus	497/497 (100%)	14.1% (70)	15.4% (45)	12.3% (25)	0.328
Coronary heart disease	493/497 (99.2%)	15.3% (76)	15.4% (45)	15.2% (31)	0.972
Dyslipidemia	495/497 (99.6%)	58.4% (290)	58.7% (172)	57.8% (118)	0.863
Previous stroke	497/497 (100%)	11.3% (56)	11.6% (34)	10.8% (22)	0.776
Pre-stroke mRS > 2	496/497 (99.8%)	8.2% (41)	7.8% (23)	8.8% (18)	0.706
*Antiplatelet therapy*	*495/497 (99.6%)*	*–*	*–*	*–*	*0.536*
None	–	67.4% (335)	69.3% (203)	64.7% (132)	–
Mono	–	30.2% (150)	28.7% (84)	32.4% (66)	–
Dual	–	2.0% (10)	1.7% (5)	2.5% (5)	–
*Anti-coagulation*	*494/497 (99.4%)*	*–*	*–*	*–*	*0.952*
None	–	87.1% (433)	86.7% (254)	87.7% (179)	–
Vitamin K antagonist	–	6.2% (31)	6.5% (19)	5.9% (12)	–
NOAC	–	6.0% (30)	6.1% (18)	5.9% (12)	–
*Other medication*	*495/497 (99.6%)*	*–*	*–*	*–*	*–*
Statin	–	25.4% (126)	26.6% (78)	23.5% (48)	0.441
*Other clinical data*					
Systolic BP, mmHg	487/497 (98.0%)	155.0 (135.0–173.0)	152.0 (134.0–171.8)	158.0 (137.0–175.0)	0.121
Diastolic BP, mmHg	488/497 (98.2%)	81.0 (71.0–95.0)	80 (70.5–95.0)	83.0 (72.0–94.0)	0.800
Admission glucose, mmol/L	488/497 (98.2%)	6.5 (5.8–7.5)	6.5 (5.7–7.7)	6.4 (5.8–7.1)	0.088
Admission NIHSS	497/497 (100%)	12 (7–18)	13 (8–18)	11 (6–17)	0.002^a^

Data are expressed as percentage (*n*) or median (interquartile range 25–75%)

*BP* blood pressure, *DWI-ASPECTS* diffusion-weighted imaging Alberta stroke program early CT score, *NIHSS* National Institutes of Health Stroke Score, *NOAC* new oral anticoagulants, *mRS* modified Rankin scale, *BP* blood pressure

^a^Statistically significant

**Table 3:** Stroke-related clinical and imaging data for the stent retriever (SR) and combined approach (COA) group

	Data available for (*n*/%)	All patients (*n* = 497)	SR ± BGC (*n* = 293)	COA ± BGC (*n* = 204)	P‑value
*TOAST*	*495/497 (99.6%)*	–	–	–	*0.564*
Large-artery atherosclerosis	–	10.7% (53)	10.6% (31)	10.8% (22)	–
Cardioembolic	–	46.9% (233)	47.1% (138)	46.6% (95)	–
Other determined causes	–	5.0% (25)	6.1% (18)	3.4% (7)	–
Undetermined	–	37.0% (184)	35.8% (105)	38.7% (79)	–
*Field strength*	*497/497 (100%)*	*–*	*–*	*10.8% (22)*	*0.788*
1.5 T	–	65.4% (325)	65.9% (193)	46.6% (95)	–
3 T	–	34.6% (172)	34.1 (100)	3.4% (7)	–
*Time to imaging/treatment*					
Time SO/LSW to admission (min)	486/497 (97.8%)	122.5 (71.0–286.0)	114.0 (69.0–268.0)	133.0 (73.0–310.0)	0.104
IV lysis prior to MRI	497/497 (100%)	6.4% (32)	5.8% (17)	7.4% (15)	0.488
IV lysis prior to MT	497/497 (100%)	38.8% (193)	37.9% (111)	40.2% (82)	0.603
Time SO/LSW to groin puncture (min)	490/497 (98.6%)	231.0 (164.8–386.5)	229.0 (164.0–372.3)	240.0 (165.8–452.0)	0.317
Time to reperfusion (min)	471/497 (94.8%)	41.0 (28.0–63.0)	36.0 (25.0–60.0)	47.0 (31.8–71.3)	0.000^a^
*Primary site of occlusion*	*497/497 (100%)*	*–*	*–*	*–*	*0.000*^a^
Intracranial ICA	–	13.9% (69)	16.0% (47)	10.8% (22)	–
MCA (M1)	–	59.8% (297)	68.9% (202)	46.6% (95)	–
MCA (M2)	–	23.7% (118)	13.7% (40)	38.2% (78)	–
MCA (M3)	–	0.6% (3)	0.0% (0)	1.5% (3)	–
MCA and ACA involved	–	1.0% (5)	1.0% (3)	1.0% (2)	–
ACA	–	1.0% (5)	0.3% (1)	2.0% (4)	–
Tandem occlusion	–	84.1% (418)	8.2% (24)	27.0% (55)	0.000^a^
*Imaging*					
DWI-ASPECTS	493/497 (99.2%)	8 (6–9)	8 (5–9)	8 (7–9)	0.020
SVS	497/497 (100%)	87.9% (437)	88.1% (258)	87.7% (179)	0.917

Data are expressed as percentage (*n*) or median (interquartile range 25–75%)

*ACA* anterior cerebral artery, *DWI-ASPECTS* diffusion-weighted imaging Alberta stroke program early CT score, *ICA* internal carotid artery, *IV* intravenous, *LSW* last seen well, *MCA* middle cerebral artery, *SO* symptom onset, *SVS* susceptibility vessel sign, *T* Tesla, *TOAST* trial of Org 10,172 in acute stroke treatment,

^a^Statistically significant

**Table 4:** Interventional and clinical outcome results for the stent retriever (SR) and combined approach (COA) group

	Data available for (*n*/%)	All patients (*n* = 497)	SR ± BGC (*n* = 293)	COA ± BGC (*n* = 204)	P‑value
*Number of passes*	*497/497 (100%)*	*–*	*–*	*–*	*0.174*
≤ 3	–	90.3% (449)	90.8% (266)	89.7% (183)	–
4–5	–	8.7% (43)	8.5% (25)	8.8% (28)	–
≥ 6	–	1.0 (5)	0.7% (2)	1.5% (3)	–
*Reperfusion/outcome*					
Final eTICI ≥ 2b	497/497 (100%)	82.9% (412)	82.3% (241)	83.8% (171)	0.647
First-pass eTICI ≥ 2b	466/497 (93.8%)	55.3% (275)	54.6% (160)	56.4% (115)	0.765
NIHSS 24 h	437/497 (87.9%)	5 (2–12)	5 (2–12)	6 (2–12)	0.837
NIHSS imp. 24 h (total)	437/497 (87.9%)	−4 (−9 to 0)	−5 (−10 to −1)	−2 (−7 to 0)	0.001^a^
NIHSS imp. 24h ≥ 4 points	437/479 (87.9%)	44.7% (222)	51.5% (151)	34.8% (71)	0.001^a^
mRS after 90 days	478/497 (98.0%)	2 (1–4)	1 (1–2)	1 (1–2)	0.596
Post-stroke mRS ≤ 2	478/497 (98.0%)	52.9% (263)	54.9% (161)	50.0% (102)	0.322
Mortality within 90 days	478/497 (98.0%)	18.7% (93)	19.1% (56)	18.1% (37)	0.825
*Complications*					
Peri-interventional complications	496/497 (99.8)	14.5% (72)	14.3% (42)	14.7% (30)	0.920
Embolization into new territory	496/497 (99.8)	3.8% (19)	3.8% (11)	3.9% (8)	0.930
Symptomatic intracerebral hemorrhage	495/497 (99.6%)	4.2% (21)	3.8% (11)	4.9% (10)	0.529

Data are expressed as percentage (*n*) or median (interquartile range 25–75%)

*eTICI* expanded thrombolysis in cerebral infarction, *mRS* modified Rankin scale, *NIHSS* National Institutes of Health Stroke Score

^a^Statistically significant

The glucose level on admission tended to be higher in patients treated with SR only than in patients treated with COA (6.5 mmol/L versus 6.4 mmol/L, *p* = 0.088). Admission NIHSS was also higher in patients treated with SR only than in those treated with COA (13 versus 11, *p* = 0.002). COA was applied more frequently in patients with tandem occlusions (27.0% versus 8.2%, *p* < 0.0001). First-line retrieval technique differed depending on the primary site of occlusion (*p* < 0.0001): SR was more often applied in patients with M1 occlusions (68.9% versus 46.6%, *p* < 0.0001) and COA was preferred for treatment of M2 occlusions (38.2% versus 13.7%, *p* < 0.0001).

### Association Between First-line Retrieval Technique and Reperfusion

There was no significant difference in FPR and overall reperfusion success between patients treated with SR only versus COA (FPR: 54.6% versus 56.4%, *p* = 0.765; overall reperfusion success: 82.3% versus 83.8%, *p* = 0.647). Time to reperfusion was shorter in patients treated with SR only than in patients treated with COA (36 min versus 47 min; *p* < 0.0001).

A multivariable binary logistic regression model that included all patients found no association between first-line SR-based retrieval technique and FPR (aOR 1.172, 95% CI 0.747–1.839; *p* = 0.490) or overall reperfusion (aOR 1.514, 95% CI 0.841–2.726; *p* = 0.167).

In another multivariable binary logistic regression model exclusively considering patients with SVS, first-line COA was not associated with FPR (aOR, 1.344, 95% CI 0.825–2.190, *p* = 0.235), but was associated with overall reperfusion (aOR 2.126, 95% CI 1.079–4.189; *p* = 0.029).

Applying the same model to patients without SVS showed that first-line SR was significantly associated with FPR (aOR 11.268, 95% CI 1.165–109.038; *p* = 0.036) and overall reperfusion success (aOR 469.212, 95% CI 2.327–94,618.855; *p* = 0.023); however, there was no interaction between SVS and retrieval technique in terms of FPR (aOR 1.736, 95% CI, 0.491–6.136; *p* = 0.392) or overall reperfusion success (aOR 3.173, 95% CI 0.752–13.387; *p* = 0.116).

**Fig. 3:**
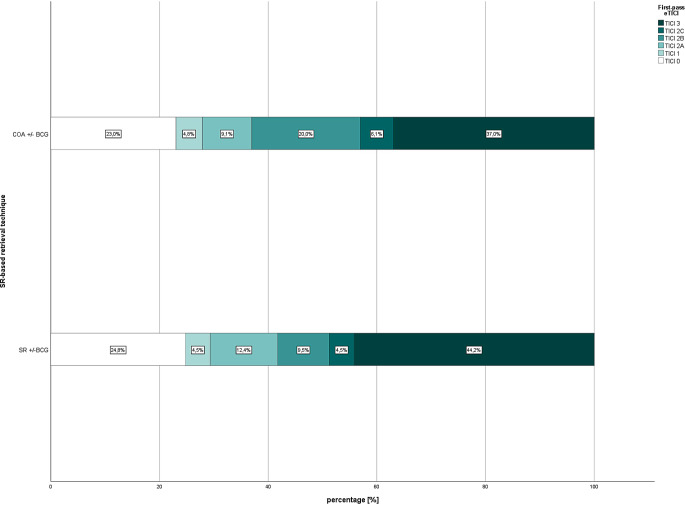
First-pass expanded treatment in cerebral infarction (eTICI) distribution according to SR-based retrieval technique for patients with acute anterior circulation stroke with susceptibility vessel sign

**Fig. 4:**
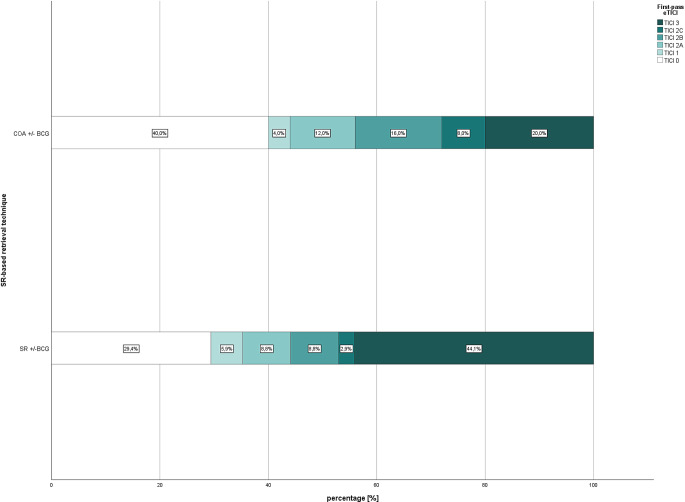
First-pass expanded treatment in cerebral infarction (eTICI) distribution according to first-line SR-based retrieval technique for patients with acute anterior circulation stroke without susceptibility vessel sign

Figures. 3 and 4 show the distribution of first-pass eTICI according to SR-based retrieval technique in patients with and without SVS. Exclusion of patients in whom a BGC was not used did not change the results regarding FPR and overall reperfusion (Supplementary Tables 7 and 8). Limiting the analyses to cases in which the same brand of stent retriever was used also did not affect results.

### Association Between First-line Retrieval Technique and Complications

First-line retrieval technique did not significantly affect the risk of periinterventional complications (14.3% versus 14.7%; *p* = 0.920), emboli in previously unaffected territories (3.8% versus 3.9%; *p* = 0.930), or symptomatic intracerebral hemorrhage (3.8% versus 4.9%; *p* = 0.568).

A multivariable binary logistic regression model that included all patients found no interaction between SVS and retrieval technique with respect to periinterventional complications (aOR 0.440, 95% CI 0.079–2.465; *p* = 0.350), emboli in previously unaffected territories (did not converge), or symptomatic intracerebral hemorrhage (aOR 0.406, 95% CI 0.024–6.748; *p* = 0.530).

These observations were unchanged when patients in whom no BGC was used were excluded (Supplementary Tables 7 and 9) or when analysis was limited to cases in which the same brand of stent retriever had been utilized.

### Clinical Outcome

Early neurological recovery was better for patients treated with SR than with COA (51.5% versus 34.8%, *p* = 0.001). On binary logistic regression analysis, SR was associated with early neurological recovery (aOR 1.936, 95% CI 1.064–3.523; *p* = 0.031); however, SVS and retrieval technique showed no significant interaction with respect to early neurological recovery (aOR 2.547, 95% CI 0.463–14.000; *p* = 0.282).

No differences were seen between groups (SR versus COA) in terms of overall mRS (1 versus 1; *p* = 0.596), functional independence (mRS ≤ 2; 54.0% versus 50.0%; *p* = 0.322), or mortality (19.1% versus 18.1%; *p* = 0.825) 90 days after treatment. These observations were unchanged when patients in whom no BGC was used were excluded (Supplementary Table 3).

## Discussion

The main findings of this study were as follows: there were no significant interactions between SR-based retrieval technique and SVS status in terms of first-pass reperfusion (1) and overall reperfusion (2). Regardless of SVS status, there were no significant differences between treatment with SR only and COA concerning the risk of periinterventional complications (3), embolization into new territories (4), or symptomatic intracerebral hemorrhage (5). Exclusion of patients in whom a BGC was not used did not change these observations.

Darcourt et al. [6] have suggested that SVS is associated with reperfusion after MT. Yet, 19–45% of AIS patients with SVS show no reperfusion after MT [5–8]. Recent randomized controlled studies have found no association between reperfusion success and second-generation thrombectomy techniques [11, 12]. This raises the question of whether an individualized strategy for MT techniques would prove more beneficial. Efforts to increase MT success by identifying imaging parameters that would allow prospective selection of the most effective retrieval technique for a specific clot are ongoing [2–4]. Bourcier et al. [4] published the first study comparing MT techniques in AIS patients with SVS and concluded that first-line SR-only thrombectomy is superior to contact aspiration among those patients [4]. Future studies will be needed to test this hypothesis prospectively (VECTOR trial; ClinicalTrials.gov Identifier: NCT04139486). Previous studies have shown that erythrocyte-rich thrombi are more likely to be apparent on SWI [13, 14] and are easier to retrieve than fibrin-rich clots [15]. Retrieving fibrin-rich clots might be more difficult because of increased vessel wall-clot interaction [16, 17]; however, this interaction, which is primarily determined by clot histology, should be similar among clots with the same SVS status. We hypothesize that differences in device-clot interaction may explain the superiority of SR-based retrieval techniques in SVS⊕ clots.

To the best of our knowledge, this is the first study to examine different SR-based retrieval techniques in AIS patients classified according to SVS status. Regardless of SVS status, our data show no superiority of any particular SR-based retrieval technique. Various factors may explain this finding. Even though a combined approach may promise synergetic effects in some cases, in others we hypothesized that the additional aspiration could also reduce the device-clot interaction by impairing or reducing the integration of the thrombus into the stent meshes. The fact that SR was performed more frequently than COA (SR ± BGC: 293 versus COA ± BGC: 204) may also play to the effect that SR was equally successful in the anterior circulation. Any experience bias in favor of SR thrombectomy could affect reperfusion success and consequently alter results [18]. Future studies could examine the efficacy of different retrieval techniques for retrieving clots with varying histological composition.

First-line SR-only treatment was associated with early neurological recovery. Several factors may have contributed to this finding, some of which were adjusted for in the regression analysis. Admission NIHSS was lower in the COA group, which might have prevented a 4-point NIHSS reduction more frequently than in patients treated with SR only. Tandem occlusions were more frequent in the COA than in the SR group. Furthermore, the speed of neurological recovery might differ depending on the primary site of occlusion. Time to reperfusion was longer for the COA group, which is probably due to a combination of factors (i.e. COA was applied more frequently in tandem and M2 occlusions, it requires positioning of two retrieval devices rather than one, and it may have been applied more frequently in cases with complex intracranial and extracranial vessel anatomy). The abovementioned experience bias in favor of SR only could also play a role here. Furthermore, microemboli/microstructural changes are not always reflected by angiographic grading scales like eTICI [19, 20]; however, our data suggest no significant interaction of SVS status and first-line SR-based retrieval technique mediating early neurological recovery.

Regardless of SVS status, the two SR-based retrieval techniques did not differ in terms of periinterventional or postinterventional complication rates; however, this does not mean that additional distal aspiration or proximal flow protection using a BGC should be avoided. Earlier studies have found that BGCs may both improve the chances of reperfusion [21, 22] and decrease the risk of ENT [23].

Despite our results, imaging biomarkers like SVS may still help to identify patients who are at high risk for futile reperfusion, or peri-interventional and postinterventional complications depending on the retrieval approach chosen. Although the binary classification of SVS does not seem to offer enough information to guide the selection of a particular stent-based retrieval technique, modern techniques like quantitative susceptibility mapping could prove beneficial by providing more specific information on clot composition [24, 25].

### Limitations

Generalizability might be limited because this was a retrospective, single center study. As suggested in a previous study and confirmed in the current analysis, baseline criteria and reperfusion outcome for stroke patients differ depending on initial imaging modality, which causes selection bias. Since SVS was first described as the gradient echo susceptibility vessel sign (GRE SVS) in T2*-weighted gradient echo imaging, most of the studies so far have not performed SWI, which provides better spatial resolution and is therefore superior in visualizing smaller clots. The better differentiation provided by SWI sequences led to an updated definition of SVS, which is independent of the contralateral vessel diameter. Both of these developments limit comparability to earlier studies [26]. Several other factors that may influence reperfusion success, such as clot density [27, 28], intracranial and extracranial vessel anatomy [29], and collateral circulation [30] could not be evaluated owing to the lack of relevant data. The choice of first-line retrieval technique was at the discretion of the treating neurointerventionalist, which is a source of selection bias. The data presented in this study were acquired over a period of 9 years during which interventional treatment standards and techniques have advanced, which may have influenced results. Angiography was not always performed after the first pass, which led to data gaps regarding first-pass eTICI and may constitute another source of selection bias. Although the stent-based retrieval techniques examined in this study (SR only ± BCG, COA ± BCG) are the only ones used at our institution and are certainly among the most frequently used worldwide, there may be other stent-based techniques that were not considered for this analysis.

## Conclusion

While previous analyses have indicated that in AIS patients with SVS, first-line SR thrombectomy may promise higher rates of reperfusion than contact aspiration, our data show no superiority of any particular SR-based retrieval technique regardless of SVS status; however, a binary classification of SVS may not provide enough information to effectively guide choice of retrieval approach. Future studies will be needed to determine whether modern techniques like quantitative susceptibility mapping allow a more nuanced selection of retrieval technique by providing more specific information on clot composition.

## Supplementary Information

Tables 1–9

## Abbreviations

AIS: Acute ischemic stroke
BGC: Balloon-guide catheter
COA: Combined approach
DSA: Digital subtraction angiography
FPR: First-pass reperfusion
MCA: Middle cerebral artery
MT: Mechanical thrombectomy
NIHSS: National Institutes of Health Stroke Scale
SR: Stent retriever
SVS: Susceptibility vessel sign
SWI: Susceptibility-weighted imaging
