# Supplementary material for: Stent-Based Retrieval Techniques in Acute Ischemic Stroke Patients with and Without Susceptibility Vessel Sign
Source: Clin Neuroradiol. 2021 Aug 31;32(2):407–18. doi: 10.1007/s00062-021-01079-1 (PMC9187552; doi:10.1007/s00062-021-01079-1)
Supplement: Supplementary file 1 — Tables 1–9 [file 62_2021_1079_MOESM1_ESM.docx]

**SUPPLEMENTARY TABLES**

| Name of scan unit | Field strength  (Tesla) | Repetition time  (ms) | Echo time  (ms) | Flip angle  (°) | Slice thickness  (mm) |
| --- | --- | --- | --- | --- | --- |
| MAGNETOM Avanto^a^ or MAGNETOM Aera^a^ | 1.5 | 49 | 40 | 15.0 | 1.6, 1.8, or 2.0 |
| MAGNETOM  Verio^a^ | 3 | 27 | 20 | 15.0 | 0 |

**Supplementary Table 1.** Technical data of SWI-sequences used to evaluate SVS status. ^a^Manufacturer: Siemens Healthcare GmbH, Erlangen, Germany

| **Device name** | **Size (in french)** |
| --- | --- |
| Sofia^TM^ Plus (MicroVention, Aliso Viejo, California, USA) | 5F,6F |
| AXS Catalyst^®^ 6 (Stryker, Kalamazoo, Michigan, USA) | 6F |
| Vasco+35 (Balt, Montmorency, France) | 5.1F |
| 3MAX Reperfusion Catheter (Penumbra, Alameda, USA) | 4.7F |
| 5MAX Reperfusion Catheter ((Penumbra, Alameda, USA) | 6F |

**Supplementary Table 2.** List of stent distal aspiration catheters devices used in this study

|  | Patients included  (n=497) | Patients excluded due to admission CT  (n=641) | P-Value |
| --- | --- | --- | --- |
| Age | 74.2 (61.9–81.8) | 74.1 (62.0–82.4) | 0.818 |
| NIHSS on admission | 12 (7–17) | 17 (13–21) | <0.001 |
| DWI-ASPECTS | 8 (6–9) | 8 (6–9) | 0.280 |
| NIHSS at 24 hours | 5 | 11 (4–18) | <0.001 |
| Successful reperfusion | 83.0% (479) | 78.2% (499/638) | 0.044 |
| Functional independence | 53.4 (308) | 29.9 (191/638) | <0.001 |
| Mortality | 18.5% (107) | 32.4% (207/638) | <0.001 |

**Supplementary Table 3.** Key results for patients with admission MRI versus admission CT. *DWI-ASPECTS* diffusion-weighted imaging Alberta stroke program early CT score, *NIHSS* National Institutes of Health Stroke Score

|  | Patients included  (n=497) | Patients excluded due to un-assessable SVS (n=37) | P-Value |
| --- | --- | --- | --- |
| Age | 74.7 (62.6–82.0) | 71.2 (61.1–79.7) | 0.337 |
| NIHSS on admission | 12 (7–18) | 15 (11– 19) | 0.121 |
| DWI-ASPECTS | 8 (6–9) | 7 (6–8) | 0.127 |
| NIHSS at 24 hours | 5 (2–12) | 6 (3.3–11) | 0.326 |
| Successful reperfusion | 82.9% (412) | 70.3% (26) | 0.952 |
| Functional independence | 52.9 (263) | 43.2% (16) | 0.466 |
| Mortality | 18.7% (93) | 24.3% (9) | 0.277 |

**Supplementary Table 4.** Key results for patients with assessable versus un-assessable SVS. *DWI-ASPECTS* diffusion-weighted imaging Alberta stroke program early CT score, *NIHSS* National Institutes of Health Stroke Score

|  | Patients included  (n=497) | Patients excluded due to posterior circulation stroke (n=44) | P-Value |
| --- | --- | --- | --- |
| Age | 74.7 (62.6–82.0) | 67.1 (57.6 –78.0) | 0.021 |
| NIHSS on admission | 12 (7–18) | 8 (5–14.7) | 0.042 |
| NIHSS at 24 hours | 5 (2–12) | 5 (1.5–12) | 0.951 |
| Successful reperfusion | 82.9% (412) | 86.4% (38) | 0.952 |
| Functional independence | 52.9 (263) | 59.1% (26) | 0.466 |
| Mortality | 18.7% (93) | 15.9% (7) | 0.277 |

**Supplementary Table 5.** Key results for patients with assessable versus un-assessable SVS.

|  | Data available for (n/[%]) | All patients  (n=362) | SR + BGC  (n=273) | COA + BGC  (n=89) | *P*-value |
| --- | --- | --- | --- | --- | --- |
| Age | 362/362 (100%) | 74.6 (63.1–82.5) | 74.2 (62.2–82.3) | 76.2 (66.6–84.2) | 0.174 |
| Sex, female | 362/362 (100%) | 51.7% | 53.5% (146) | 46.1% (41) | 0.224 |
| Risk factors |  |  |  |  |  |
| Hypertension | 362/362 (100%) | 62.7% (227) | 35.2% (96) | 56.2% (50) | 0.143 |
| Smoking | 361/362 (99.7%) | 27.6% (100) | 27.8% (76) | 27.0% (24) | 0.858 |
| Diabetes mellitus | 362/362 (100%) | 13.5% (49) | 13.9% (38) | 12.4% (11) | 0.709 |
| Dyslipidemia | 361/362 (99.7%) | 58.0% (210) | 57.5% (157) | 59.6% (53) | 0.761 |
| Previous stroke | 362/362 (100%) | 11.6% (42) | 12.1% (33) | 10.1% (9) | 0.613 |
| Pre-stroke mRS>2 | 361/362 (99.7%) | 8.0% (29) | 8.4% (23) | 6.7% (6) | 0.606 |
| Antiplatelet therapy | 360/362 (99.4%) |  |  |  | 0.781 |
| None |  | 67.4% (244) | 68.5% (187) | 64.0% (57) |  |
| Mono |  | 30.1 (109) | 29.3% (80) | 32.6% (29) |  |
| Dual |  | 1.9% (7) | 1.8% (5) | 2.2% (2) |  |
| Anti-coagulation | 359/362 (99.2%) |  |  |  | 0.629 |
| None |  | 87.3% (316) | 86.4% (236) | 89.9% (80) |  |
| Vitamin K-antagonist |  | 6.1% (22) | 6.6% (18) | 4.5% (4) |  |
| NOAC |  | 5.8% (21) | 6.2% (17) | 4.5% (4) |  |
| Other medication | 361/362 (99.7%) |  |  |  |  |
| Statin |  | 24.3% (88) | 26.7% (73) | 16.9% (15) | 0.057 |
| Other clinical data |  |  |  |  |  |
| Systolic BP, mmHG | 354/362 (97.8%) | 151.5 (134.0–170.0) | 150.5 (133.3 –170.0) | 156.0 (136.5 –174.0) | 0.247 |
| Diastolic BP, mmHG | 355/362 (98.1%) | 81.0 (71.0–95.0) | 80.0 (70.0–95.0) | 85.0 (74.8–96.0) | 0.193 |
| Admission glucose, mmol/L | 356/362 (98.3%) | 6.5 (5.8–7.5) | 6.5 (5.7–7.6) | 6.5 (5.9–7.1) | 0.685 |
| Admission NIHSS | 362/362 (100%) | 13 (8–18) | 14 (8–18) | 13 (7–17.5) | 0.310 |
| TOAST | 360/362 (99.4%) |  |  |  | 0.259 |
| Large-artery atherosclerosis |  | 10.5% (38) | 11.0% (30) | 9.0% (8) |  |
| Cardioembolic (CE) |  | 44.5% (161) | 46.2% (126) | 39.3% (35) |  |
| Other determined causes |  | 5.5% (20) | 6.2% (17) | 3.4% (3) |  |
| Undetermined |  | 39..0% (141) | 36.3% (99) | 47.2% (42) |  |
| Field strength | 362/362 (100%) |  |  |  | 0.274 |
| 1.5 Tesla |  | 66.0% (239) | 64.5% (176) | 70.8% (63) |  |
| 3 Tesla |  | 34.0% (123) | 35.5% (97) | 29.2% (26) |  |
| Time to imaging/  treatment |  |  |  |  |  |
| Time SO/LSW to admission (min) | 357/362 (100%) | 116.0 (70.0–275.5) | 110.0 (69.3–265.0) | 126.0 (70.5–329.0) | 0.257 |
| IV lysis prior to MRI | 362/362 (100%) | 8.3% (30) | 5.9% (16) | 15.7% (14) | 0.003^*^ |
| IV lysis prior MT | 362/362 (100%) | 42.0% (152) | 38.8% (106) | 51.7% (46) | 0.033^*^ |
| Time SO/LSW to groin puncture (min) | 358/362 (98.9%) | 230 (165.8–373.3) | 222.0 (163.0–362.5) | 254.0 (175.5–467.0) | 0.124 |
| Time to recanalization (min) | 344/362 (95.0%) | 40.0 (25.3–63.0) | 36 (23.3–55.8) | 55.5 (38.3–79.8) | 0.000^*^ |
| Primary site of occlusion | 362/362 (100%) |  |  |  | 0.000^*^ |
| Intracranial ICA |  | 15.2% (55) | 16.1% (44) | 12.4% (11) |  |
| MCA (M1) |  | 66.0% (239) | 70.0% (191) | 53.9% (48) |  |
| MCA (M2) |  | 16.3% (59) | 12.5% (34) | 28.1% (25) |  |
| MCA and ACA involved |  | 1.1% (4) | 1.1% (3) | 1.1% (1) |  |
| ACA |  | 1.4% (5) | 0.4% (1) | 4.5% (4) |  |
| Tandem occlusion | 362/362 (100%) | 11.6% (42) | 4.8% (13) | 32.6% (29) | 0.000^*^ |
| Imaging |  |  |  |  |  |
| DWI-APECTS | 358/362 (98.9%) | 8 (6–9) | 8 (5–9) | 8 (7–9) | 0.173 |
| SVS | 362/362 (100%) | 87.3% (316) | 87.5% (239) | 86.5% (77) | 0.800 |

**Supplementary Table 6.** Baseline characteristics and stroke-related data for the stent retriever (SR) with balloon-guided catheter (BCG) and combined approach (COA) with BCG group. Data are expressed as percentage (n) or mean (interquartile range 25–75%). *ACA* anterior cerebral artery, *BP* blood pressure, *DWI-ASPECTS* diffusion-weighted imaging Alberta stroke programme early CT score, *ICA* internal carotid artery, *IV* intravenous, *LSW* last seen well, *MCA* middle cerebral artery, *NOAC* new oral anticoagulants, *SO* symptom onset, *SVS* susceptibility vessel sign, *TOAST* trial of Org 10172 in acute stroke treatment (TOAST). ^*^statistically significant

| Number of passes | 362/362 (100%) |  |  |  | 0.114 |
| --- | --- | --- | --- | --- | --- |
| ≤ 3 |  | 90.6% (328) | 91.2% (249) | 88.8% (79) |  |
| 4–5 |  | 8.3% (30) | 8.1% (22) | 9.0% (8) |  |
| ≥ 6 |  | 1.1% (4) | 0.7% (2) | 2.2% (2) |  |
| Recanalization/outcome |  |  |  |  |  |
| Final eTICI ≥2b | 362/362 (100%) | 84.0% (304) | 84.6% (231) | 82.0% (73) | 0.563 |
| First-pass eTICI ≥2b | 339/362 (93.6%) | 55.0% (199) | 55.3% (151) | 53.9% (48) | 0.797 |
| NIHSS 24h | 325/362 (89.7%) | 6 (2–12) | 5 (2–12) | 8 (3–14) | 0.056 |
| NIHSS improvement 24h (total) | 325/362 (89.7%) | −4 (−9 to −1) | −5 (−10 to −1) | −2.5 (−7.3 to 0) | 0.009^*^ |
| NIHSS improvement 24h ≥4 points | 325/362 (89.7%) | 48.1% (174) | 52.7% (144) | 33.7% (30) | 0.002^*^ |
| mRS after 90 days | 347/362 (95.9) | 2 (1–4) | 2 (1–4) | 3 (1–4) | 0.082 |
| Post-stroke mRS≤2 | 347/362 (95.9) | 53.0% (192) | 55.3% (151) | 46.1% (41) | 0.167 |
| Mortality within 90 days | 347/362 (95.9) | 18.5% (67) | 18.7% (51) | 18.0% (16) | 0.945 |
| Complication |  |  |  |  |  |
| Peri-interventional complications | 361/362 (99.7%) | 15.2% (55) | 14.3% (233) | 18.0% (16) | 0.407 |
| Embolization into new territory | 361/362 (99.7%) | 4.1% (15) | 4.0% (11) | 4.5% (4) | 0.853 |
| Symptomatic intracerebral hemorrhage | 360/362 (99.4%) | 4.7% (17) | 3.7% (10) | 7.9% (7) | 0.100 |

**Supplementary Table 7.** Interventional and clinical outcome results for the stent retriever (SR) + balloon-guided catheter (BCG) and combined approach (COA) + BCG group. Data are expressed as percentage (n) or mean (interquartile range 25–75%). *eTICI* expanded thrombolysis in cerebral infarction, *mRS* modified Rankin scale, *NIHSS* National Institutes of Health Stroke Scale.^*^statistically significant

| Population examined | Outcome variable | aOR | 95% CI lower | 95% CI upper | P-value |
| --- | --- | --- | --- | --- | --- |
| All patients | First-pass reperfusion | 1.032 | 0.574 | 1.856 | 0.917 |
|  | Overall reperfusion | 0.783 | 0.369 | 1.659 | 0.523 |
| SVS+ patients | First-pass reperfusion | 1.319 | 0.688 | 2.530 | 0.405 |
|  | Overall reperfusion | 1.445 | 0.574 | 3.639 | 0.435 |
| SVS− patients | First-pass reperfusion | 0.108 | 0.006 | 1.895 | 0.128 |
|  | Overall reperfusion | 0.135 | 0.014 | 1.311 | 0.084 |

**Supplementary Table 8.** Association between SR-based retrieval technique and reperfusion excluding patients in whom balloon-guided catheters were not utilized**.** *aOR* adjusted odds ratio, *CI* confidence interval

| Population examined | Outcome variable | aOR | 95% CI lower | 95% CI upper | P-value |
| --- | --- | --- | --- | --- | --- |
| All patients | Peri-interventional complications | 0.262 | 0.039 | 1.746 | 0.166 |
|  | Embolization into new territorries | ^a^ | ^a^ | ^a^ | ^a^ |
|  | Symptomatic intracerebral hemorrhage | 0.775 | 0.050 | 11.883 | 0.855 |

**Supplementary Table 9.** Association between SR-based retrieval technique and peri- and postinterventional complication parameters excluding patients in whom balloon-guided catheters were not utilized. All data relate to the interaction variable of SR-based retrieval technique and SVS. *aOR* adjusted odds ratio, *CI* confidence interval, ^a^calculation did not converge
